# Inhibitory effect of β-escin on Zika virus infection through the interruption of viral binding, replication, and stability

**DOI:** 10.1038/s41598-023-36871-1

**Published:** 2023-06-20

**Authors:** Zheng-Zong Lai, Hsin-Hsuen Shen, Yen-Mei Lee

**Affiliations:** 1 Graduate Institute of Medical Science, National Defense Medical Center, Taipei, 114 Taiwan; 2 Department and Graduate Institute of Pharmacology, National Defense Medical Center, Taipei, 114 Taiwan; 3 Department of Pharmacy Practice, Tri-Service General Hospital, National Defense Medical Center, Taipei, 114 Taiwan

**Keywords:** Virology, Drug discovery, Microbiology

## Abstract

β-Escin is a mixture of triterpenoid saponins extracted from horse chestnut seeds that have diverse pharmacological activities, including anti-inflammation, anti-edematous, venotonic, and antiviral effects. In the clinical setting, β-escin is primarily used to treat venous insufficiency and blunt trauma injuries. The anti-Zika virus (ZIKV) activity of β-escin has not been explored. This study investigated the antiviral efficacy of β-escin on ZIKV and dengue virus (DENV) in vitro and then elucidated the underlying mechanism. The inhibitory effects of β-escin on viral RNA synthesis, protein levels, and infection ability were determined using qRT-PCR, Western blotting, and immunofluorescence assays, respectively. To further characterize how β-escin interferes with the viral life cycle, the time-of-addition experiment was performed. An inactivation assay was performed to determine whether β-escin affects ZIKV virion stability. To broaden these findings, the antiviral effects of β-escin on different DENV serotypes were assessed using dose-inhibition and time-of-addition assays. The results showed that β-escin exhibits anti-ZIKV activity by decreasing viral RNA levels, protein expression, progeny yield, and virion stability. β-Escin inhibited ZIKV infection by disrupting viral binding and replication. Furthermore, β-escin demonstrated antiviral activities against four DENV serotypes in a Vero cell model and prophylactic protection against ZIKV and DENV infections.

## Introduction

Zika virus (ZIKV), a member of the genus *flavivirus* of the family *Flaviviridae*, is an arbovirus primarily transmitted by infected mosquitoes. Over the past two decades, there had been several large ZIKV outbreaks. One occurred in 2007 on the Western Pacific Island of Yap in the Federated States of Micronesia^1^, another occurred in French Polynesia in the South Pacific in 2013–2014^2^, and yet another happened in Central and South American countries and West Africa in 2015–2016^3^. Following these events, the number of infectious cases decreased in recent years. A study by the WHO revealed that 87 countries are known to have native mosquito-borne ZIKV^4^, indicating that the threat of ZIKV infection remains a concern. Preventive measures such as avoiding mosquito bites and preventing travelling to anywhere that ZIKV is spreading are suggested.

In addition to mosquito bites, ZIKV can be spread through other routes, such as blood transfusion^5^, sexual intercourse^6^, and maternal-to-fetal vertical infection^7^. These multiple infection routes make it a difficult public health issue and suggest that the next outbreak may not be limited only to tropical countries.

Infection with ZIKV may result in asymptomatic or mild symptoms including fever, rash, muscle pain, and conjunctivitis^8^. People with mild symptoms should get plenty of rest, drink fluids, and treat symptoms with antipyretics such as acetaminophen to ease pain and fever. If symptoms worsen, patients should seek medical care. Some severe neurological disorders, such as Guillain–Barré syndrome in adults^9,10^ and congenital Zika syndrome in infants^11^, are strongly linked to ZIKV infection. ZIKV-induced congenital birth defects include microcephaly, intracranial calcifications^12^, hearing deficits, and intrauterine growth restriction^13,14^, which shows that ZIKV possesses unique pathological features that are different from other flaviviruses.

Based on the evidence, ZIKV-induced medical problems require more attention and novel treatment options. Progress in anti-ZIKV drug discovery and development has been advanced in the last few years; however, approved drugs are not yet available. Identifying new compounds or repurposing current drugs could enhance the pipeline of antiviral drugs. Natural or herbal compounds are abundant and an excellent resource to discover novel antiviral drugs. They also have the advantage of exhibiting stable physicochemical activities^15^.β-Escin or aescin, the major active component of horse chestnut seeds (*Aesculus hippocastanum* L.), is a primary mixture composed of escin Ia and escin Ib^16^. β-Escin is traditionally used to treat chronic venous insufficiency^17^. It also exhibits ameliorating effects on edema^18^, diabetes^19^, obesity^20^, inflammation^21–23^, and cancer^24–26^. Multiple mechanisms of β-escin activity have been reported previously, such as raising venous tension via enhancement of calcium ion entry, increasing the release of prostaglandin F2α^27,28^, and antagonism to serotonin and histamine^29^. The anti-inflammatory activity of β-escin is associated with its inhibitory effects on hyaluronidase^30^, NF-κB, and TNF-α^31^.

Recently, β-escin has been found to exert antiviral effects on porcine epidemic diarrhea virus (PEDV), herpes virus 1 (HSV-1), respiratory syncytial virus (RSV), and dengue virus-2 (DENV-2)^32–34^. Since ZIKV and DENV-2 belong to the same genus, i.e., flavivirus, we assessed whether β-escin exhibits anti-ZIKV efficacy in vitro and explored the putative underlying mechanism. The inhibitory effects of β-escin on four different serotypes of DENV in vitro were also evaluated.

## Results

### β-Escin exhibits mild cytotoxicity against a normal cell line

**Figure 1:**
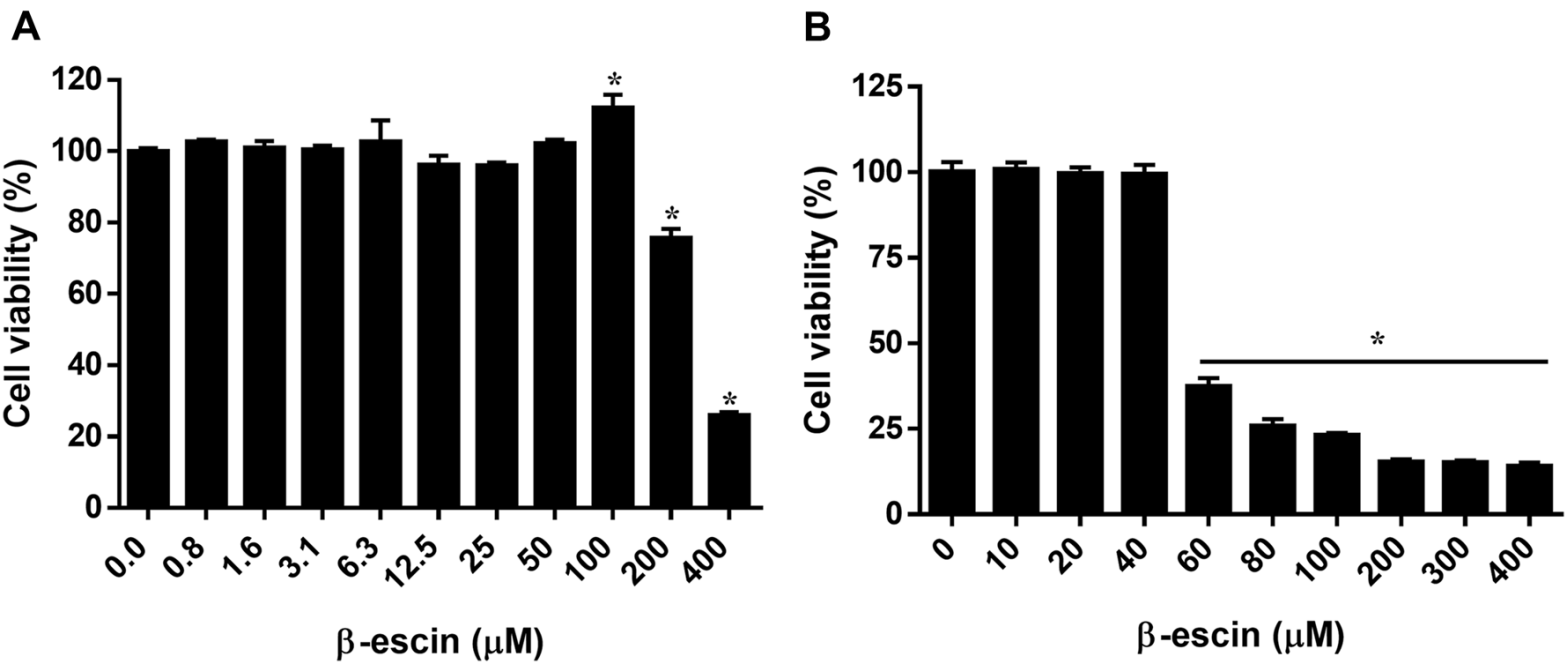
Cytotoxicity of β-escin in vitro. The cytotoxicity of β-escin-treated (**A**) Vero cells or (**B**) A549 cells for 30 h was determined using CCK-8 assays. The results showed that β-escin was non-cytotoxic in both Vero and A549 cell lines at a concentration within 40 μM. Data represent the mean ± standard deviation of at least three independent experiments. *p < 0.05 vs. control group.

First, we assessed the cytotoxicity of β-escin in Vero cells (African green monkey kidney cells) and A549 cells (adenocarcinoma human alveolar basal epithelial cells) as these 2 cells are permissive to ZIKV. This established an optimal concentration range for subsequent experiments. Over a 30-h incubation period with various concentrations of β-escin (0–400 μM), β-escin significantly inhibited Vero cell viability at concentrations over 200 μM as determined by the CCK-8 assay (Fig. 1A). Below 50 μM concentrations, cell viability was not affected; however, β-escin increased cell proliferation at concentrations up to 100 μM. The 50% cytotoxicity concentration (CC_50_) values for β-escin were over 200 μM for Vero cells. These results indicate that β-escin exhibits mild cytotoxicity.

When the assay was performed in A549 human lung cancer cells at concentrations greater than 60 μM, cell viability significantly decreased (Fig. 1B). The significant cytotoxicity of β-escin against lung cancer cells has been previously reported^35^. To avoid β-escin-induced cytotoxicity in subsequent antiviral experiments, the concentration was limited to within 30 μM in both Vero cells and A549 cells.

### β-Escin shows antiviral activities against ZIKV infection

**Figure 2:**
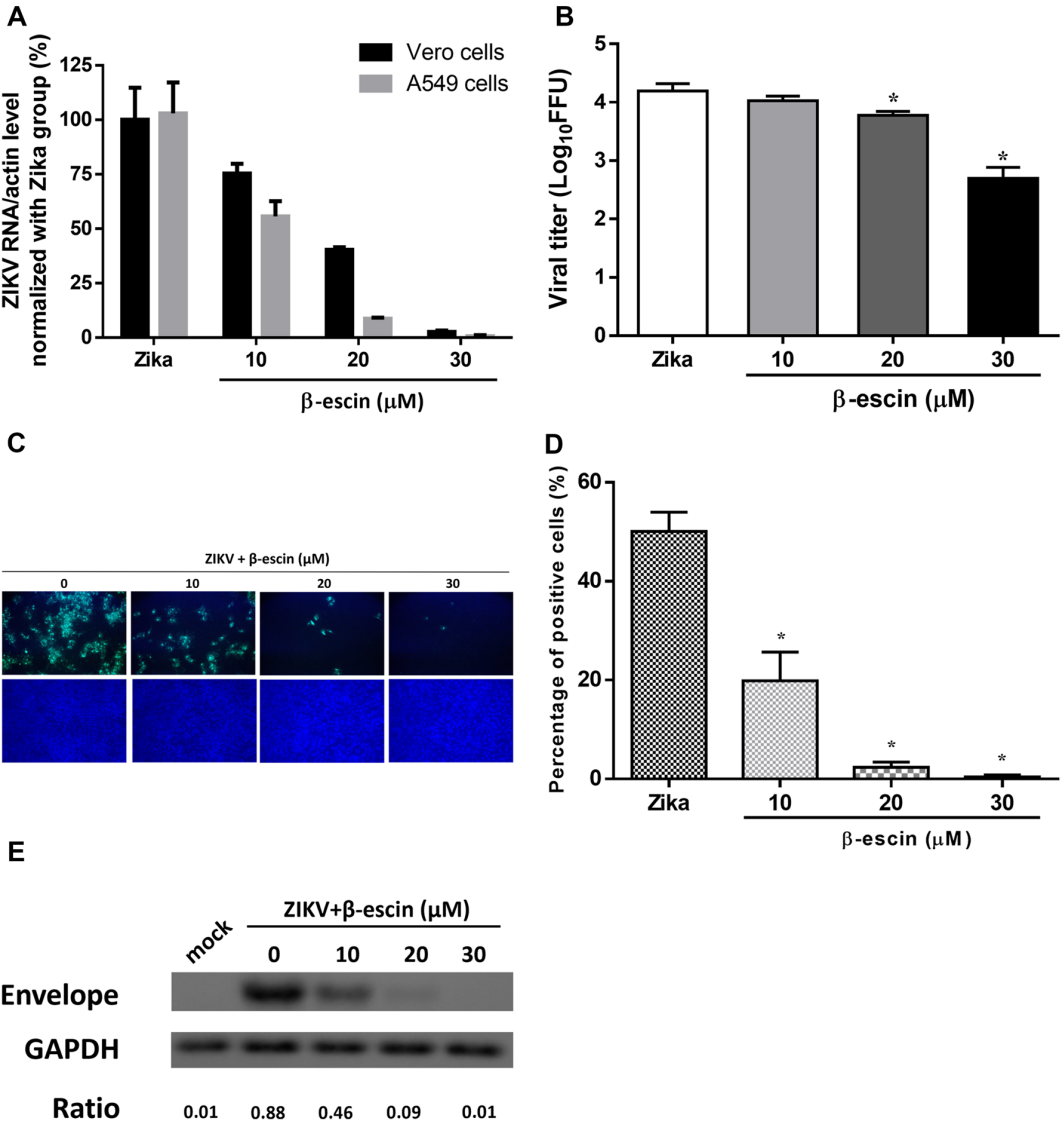
Antiviral effects of β-escin on ZIKV infection in vitro. (**A**) After 30 h of β-escin treatment, the viral RNA levels of ZIKV-infected Vero cells or A549 cells (MOI = 0.02) were determined by qRT-PCR. (**B**) The virus titers of supernatants in Vero cells were determined using the fluorescent focus assay (FFA). (**C**) The inhibitory effects of β-escin in ZIKV-infected Vero cells were examined using the immunofluorescence assay (IFA). (**D**) Percentage of ZIKV-infected Vero cells was determined using ImageJ software. (**E**) The viral protein envelope was detected by Western blotting. The results showed that β-escin was effective in inhibiting ZIKV infection. Data represent the mean ± standard deviation of at least three independent experiments. *p < 0.05 vs. Zika group.

To examine the antiviral effect and inhibitory activity of β-escin, we carried out a dose-inhibition assay in infected cells. Vero or A549 cells were seeded into 12-well plates overnight and then infected with ZIKV at a multiplicity of infection (MOI) of 0.02 along with various concentrations of β-escin for 30 h. After 30-h incubation, total RNA was extracted and viral RNA levels were measured by qRT-PCR. For both cell lines, the results indicated that β-escin inhibited ZIKV RNA levels in a dose-dependent manner (Fig. 2A). Notably, viral RNA levels were suppressed by nearly 98% at a concentration of 30 μM in both cell lines.

Further analysis of viral yield collected from the supernatants of Vero cells revealed that the titers of viral progeny were decreased in a dose-dependent manner (Fig. 2B). Next, an IFA and Western blot assay were performed to verify the inhibitory effects of β-escin on infected Vero cells. The results indicated that β-escin limited ZIKV infection and reduced viral envelope protein expression (Fig. 2C–E). Overall, these findings indicate that β-escin is effective in inhibiting ZIKV infection.

### β-Escin reduces ZIKV RNA at the early and late stages of infection

**Figure 3:**
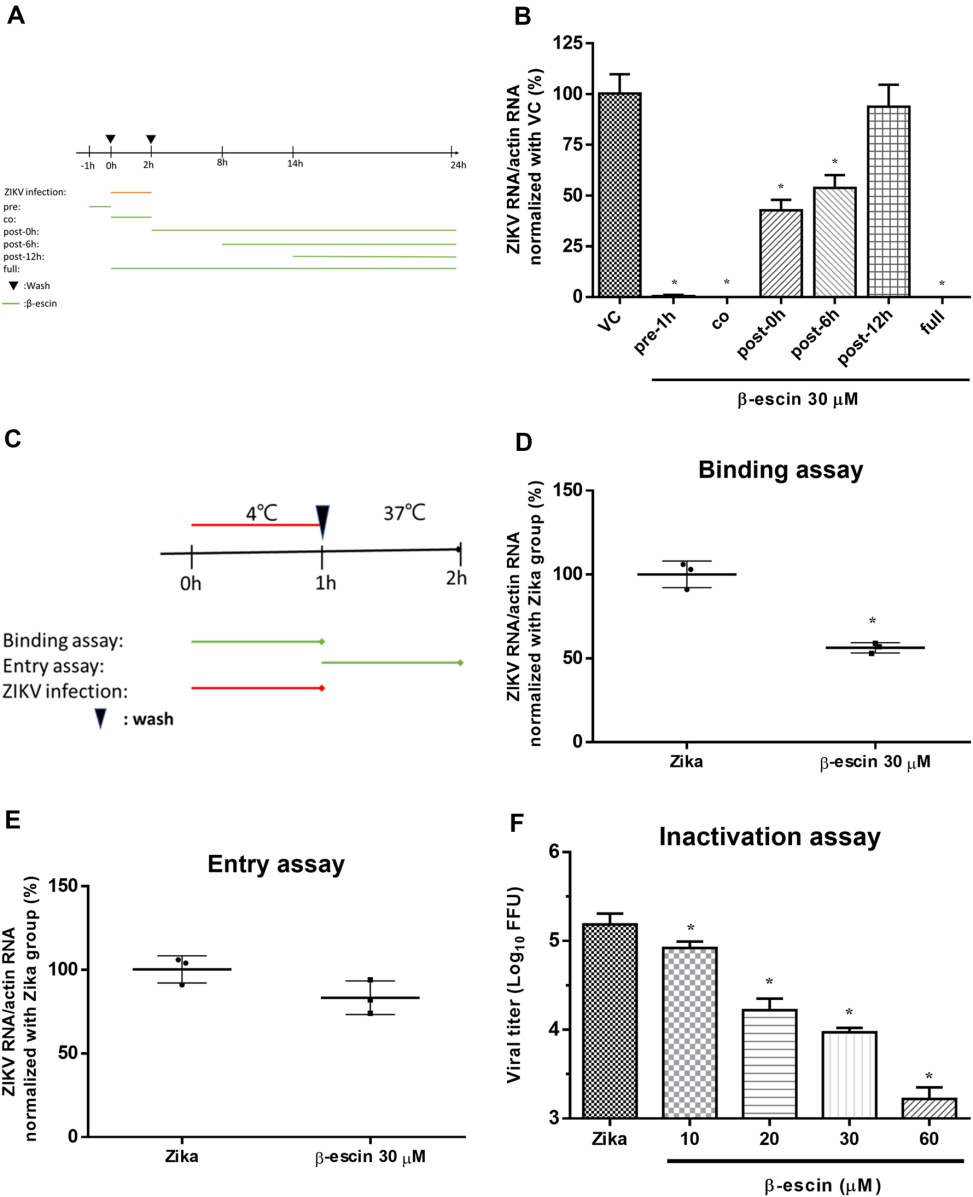
Underlying mechanism of anti-Zika virus (ZIKV) infection. (**A**) Timeline of the time-of-addition. (**B**) Time-of-addition assay: 30 μM of β-escin was added to the infected cells (MOI = 0.2) at distinct time points relative to the infection period. After 24 h, the viral RNA levels were measured by qRT-PCR. The results showed that β-escin inhibited ZIKV at the early and late life cycle stages and causes prophylactic inhibition. (**C**) Timeline of binding and entry assays. (**D**) Binding assay. Virus and β-escin were simultaneously added in the cells at 4 °C for 1 h, and then viral RNA levels were determined by qRT-PCR. (**E**) Entry assay. Cells were initially infected with virus at 4 °C for 1 h, and then adding β-escin in the infected cells at 37 °C for another 1-h. The viral RNA levels were determined by qRT-PCR. The results confirmed the inhibitory effects of the compound during the binding stage, rather than the entry stage. (**F**) Inactivation assay. In the cell-free incubation assay, ZIKV stock (2 × 10^6^ FFU) was mixed with various concentrations of β-escin at 37 °C for 2 h. The drug-virus mixtures were first diluted tenfold and, subsequently, the FFA assay was done to assess virion stability. This result confirms that β-escin can directly reduce virion stability. Data represent the mean ± standard deviation of at least three independent experiments. *p < 0.05 vs. Zika group.

To further characterize how β-escin interferes with the ZIKV life cycle, time-of-addition assays were performed. Vero cells were infected with ZIKV (MOI = 0.2) for 2 h and 30 μM β-escin was added at different time points relative to the infection. The time-of-drug addition assay was performed for seven groups: virus control (VC), pre-treatment 1 h (pre-1 h), co-treatment (co), post-treatment 0 h (post-0 h), post-treatment 6 h (post-6 h), post-treatment 12 h (post-12 h), and full-treatment (Fig. 3A). At 24 h p.i., ZIKV RNA levels were measured by qRT-PCR. The VC and full-treatment groups represented negative and positive controls, respectively (Fig. 3B). ZIKV RNA levels were significantly decreased in the pre-treatment, co-treatment, and post-treatment groups compared with the VC group, indicating that β-escin inhibited ZIKV at the early and late life cycle stages and causes prophylactic inhibition.

To further elucidate the inhibitory effects of β-escin in the co-treatment group, binding and entry assays were performed. The results confirmed the inhibitory effects of the compound during the binding stage, rather than the entry stage (Fig. 3C–E).

For the post-treatment groups (post-0 h and post-6 h), the RNA levels were gradually less inhibited with increasing time (Fig. 3B), which further indicates the compound’s inhibitory effect on viral replication.

Taken together, these findings indicate that β-escin exhibits anti-ZIKV activity by interfering with multiple stages of the viral life cycle, including blockage of binding and replication. Furthermore, β-escin exerts prophylactic protection against ZIKV infection.

### β-Escin interferes with ZIKV virion stability

Next, we used an inactivation assay to determine whether β-escin affects ZIKV virion stability. In the cell-free incubation assay, ZIKV stock (2 × 10^6^ FFU) was mixed with various concentrations of β-escin at 37 °C for 2 h. The drug-virus mixtures were first diluted tenfold and, subsequently, the FFA assay was done to assess virion stability. The results showed that the compound reduced virus titer during the assay (Fig. 3F), which further confirms that β-escin can directly reduce virion stability. The putative antiviral mechanism of β-escin was summarized in Fig. 6.

### β-Escin inhibits four serotypes of dengue virus

**Figure 4:**
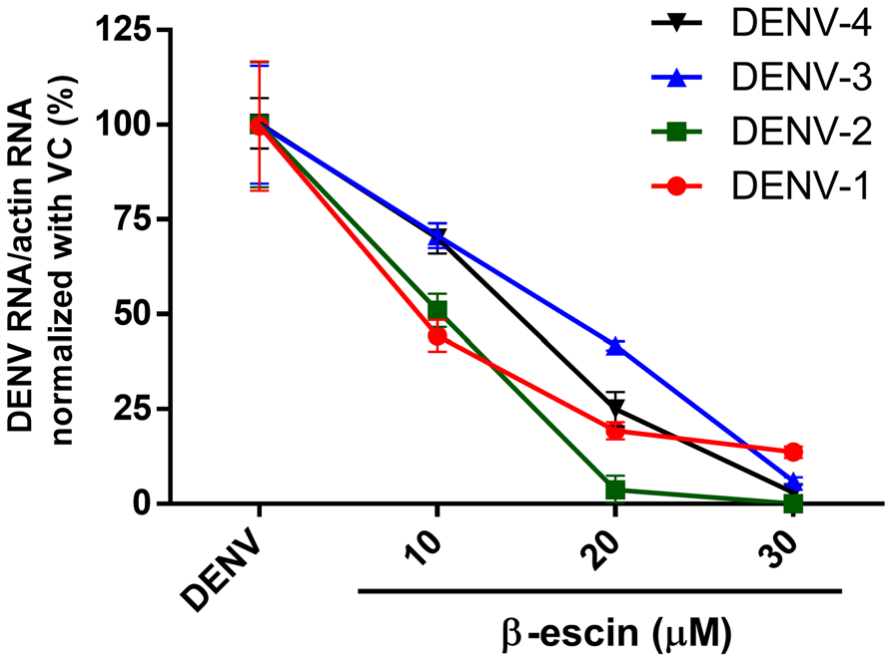
β-Escin inhibited all four serotypes of dengue virus (DENV) infection. Infected Vero cells (MOI = 1) were treated with β-escin for 60 h. The intracellular viral RNA levels were then determined by qRT-PCR. The results indicated that all four serotypes of DENV RNA were decreased following treatment with β-escin. Data represent the mean ± standard deviation of at least three independent experiments.

A previous study reported that β-escin inhibited DENV-2 infection^32^. We broadened our study to examine the antivirus ability of β-escin on different serotypes of DENV. Distinct DENV serotype-infected Vero cells (MOI = 1) were treated with indicated concentrations of β-escin for 60 h. The qRT-PCR results indicated that all four serotypes of DENV RNA were decreased following treatment with β-escin (Fig. 4).

**Figure 5:**
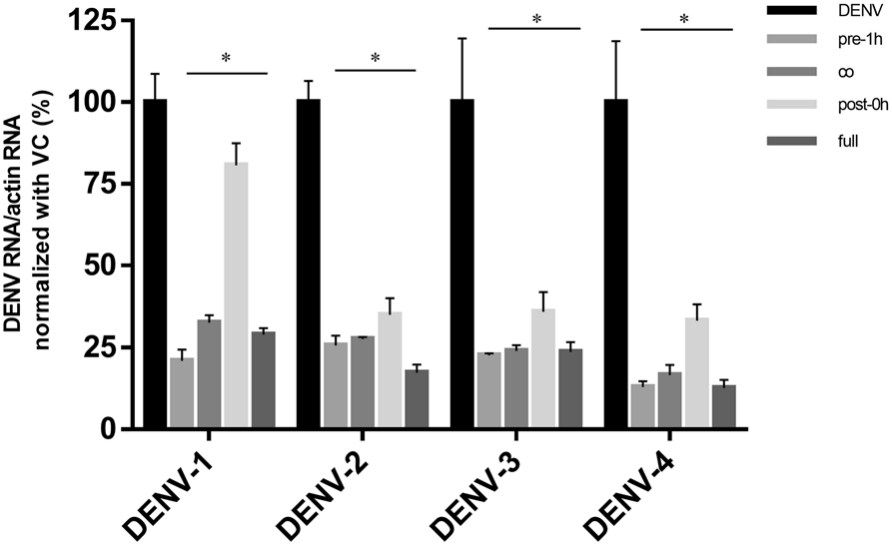
Time-of-addition assay of β-escin on DENV infection. Four serotypes of dengue virus (DENV)-infected Vero cells (MOI = 1) were treated with β-escin (30 μM) at distinct time points of infection and incubated for 40 h. The intracellular viral RNA levels were determined by qRT-PCR. The results showed that β-escin inhibited DENV at the early and late life cycle stages and causes prophylactic inhibition. Data represent the mean ± standard deviation of at least three independent experiments. *p < 0.05 vs. virus group.

Furthermore, the results of the time-of-addition experiment indicated that β-escin decreased all four serotypes of DENV in the pre-treatment 1 h, co-treatment, and post-treatment 0 h groups (Fig. 5), further demonstrating the compound’s antiviral activity in the early and late stages of DENV infection and its prophylactic anti-DENV activity. Taken together, these findings suggest that β-escin has potential application value for treating DENV infection.

## Discussion

Clinical formulations of β-escin are available as an oral tablet and transdermal gel. A meta-analysis found that oral β-escin preparations are well-tolerated and exhibit few side effects^36^, which is consistent with our findings of its low cytotoxicity in Vero cells (Fig. 1A). β-Escin also exhibits anticancer effects in lung adenocarcinoma^25^, which is consistent with our observation of higher cytotoxicity in lung cancer A549 cells (Fig. 1B). Using permissive cells for screening antiviral drug study is essential because a virus is a microorganism that needs to infect a cell as host for virus growth and replication. Besides, cellular tropism is one of the major characteristics of a virus to maintain a successful infectious cycle in target cells. If a cell line we adopt is non-permissive for virus infection, it is difficult to evaluate the amount of virus in the cells when a compound is added.

In Fig. 1A, the cell viability of Vero cells treated with 100 μM β-escin was higher than other concentration groups. For this, we speculate that β-escin may have proliferation potential because the compound was found to facilitate muscle regeneration in a rat model of skeletal muscle injury^37^. This putative relationship warrants further research in future.

In the first study of the antiviral effect of β-escin in 1999, β-escin was reported to inhibit HIV-1 protease^34^. Subsequently, β-escin was revealed to inhibit HIV by inactivating HIV particles and modulating the distinct immune response, such as downregulating HIV-1-induced NF-κB and AP-1 activation in macrophages, but upregulating NF-κB and AP-1 activation in epithelial cells^32^. While the former Michelini research team showed that β-escin possessed anti-DENV-2 effects, they did not explore the possible mechanism. β-Escin can inhibit respiratory syncytial virus (RSV) by inactivating RSV particles and suppressing RSV-induced NF-κB and AP-1 activation in macrophages and epithelial cells^33^. Previous reports have shown that compounds with a structure similar to β-escin can suppress severe acute respiratory syndrome-coronavirus (SARS-CoV) activity^38^. It thus appears that β-escin has broad-spectrum antiviral activity. In the present study, we proved that β-escin possesses antiviral activity against ZIKV and DENV in vitro.

In the context of ZIKV, β-escin inhibited the virus by reducing viral RNA (Fig. 2A), protein expression (Fig. 2E), progeny yields (Fig. 2B), and viral infectivity (Fig. 2C,D). The time-of-addition experiment results proved that β-escin interfered with the early and late stages of ZIKV infection (Fig. 3B). The addition of β-escin during the pre-treatment stage reduced virus growth, suggesting it may act on host cells by blocking cell receptors or modulating the immune response, such as NF-κB and AP-1^33^. This part of the putative mechanism warrants further research.

The addition of β-escin during the co-treatment stage also reduced virus growth, suggesting that it may act on viral particles or block virus-to-cell interactions. Based on the inactivation assay, binding and entry assay results, β-escin was proven to directly inactivate viral particles (Fig. 3F) and to stop ZIKV from binding to the cells in the binding assay rather than in the entry step (Fig. 3D,E). For the binding assay, virus and β-escin were simultaneously added in the cells at 4 °C for 1 h. At 4 °C, the virus was allowed to bind to the surface of cells but was restricted to enter cells. For the entry assay, cells were initially infected with the virus at 4 °C for 1 h, and then adding β-escin in the infected cells at 37 °C for another 1-h. Thus, whether β-escin blocks viral binding or entry can be observed by determining viral RNA levels using qRT-PCR assay.

The viral envelope (E) protein of ZIKV plays an important role during the flavivirus binding course. First, the E protein binds to glycosaminoglycans, such as heparan sulfate, and then contributes to high-affinity receptor binding^39^. Among them, C-type lectin DC-SIGN mediates the interaction^40^ and, subsequently, clathrin-mediated endocytosis occurs^41^. We speculate that the inactivation of β-escin on ZIKV virion may result from the perturbation of membrane integrity. The virucidal activity of β-escin was found for other enveloped viruses in addition to ZIKV, such as DENV, VSV and HSV, but β-escin was unable to affect non-enveloped virus stability, such as adenovirus^32,33^. This suggests that β-escin may possess a particular inhibition on viruses with envelope structure.

The addition of β-escin during the post-treatment stage also reduced viral growth, which suggests that β-escin interferes with post-entry events such as viral RNA synthesis, protein expression, assembly, and release. The administration of β-escin caused a significant reduction of RNA levels at 0 h p.i. and 6 h p.i., but not at 12 h p.i. (Fig. 3B), further implying that β-escin fits a feature of viral replication inhibitor. Previous studies reveal that β-escin can induce autophagy in human osteosarcoma cells in vitro and in vivo^42^, and the activation of autophagy blocks ZIKV infection^43^. Thus, the inhibitory mechanism of β-escin on ZIKV replication may be associated with the up-regulation of autophagy. Previous studies have also shown that β-escin decreases TNFα-induced NF-κB activation in endothelial cells^44^ and reduces NF-κB activation in HSV-infected macrophages^32^. NF-κB activation is known to increase during ZIKV infection^43^; therefore, the inhibitory effects of β-escin on ZIKV replication may be associated with the down-regulation of the NF-κB pathway. Further research is warranted to clarify these putative mechanisms. Overall, this study has demonstrated that the inhibitory effects of β-escin occur through effects on ZIKV binding, replication, and stability.

For DENV, previous research has only shown that β-escin inhibits DENV-2. This work has broadened the known antiviral range by proving that β-escin can inhibit four serotypes of DENV (Fig. 4). Furthermore, inhibition occurred during the early and late stages of infection (Fig. 5), which is a similar trend as observed for ZIKV.

## Conclusions

Taken together, the results display that β-escin inhibits ZIKV and DENV infection in a dose-dependent manner without cytotoxicity. β-escin acts as an anti-ZIKV agent by blocking viral binding, replication, and virion stability. β-Escin possesses antiviral activities against ZIKV and four serotypes of DENV at the early and late life cycle stages and causes prophylactic inhibition. These findings suggest that β-escin has potential application value for treating flaviviruses infection.

## Methods

### Cells, viruses, and chemical compounds

Vero cells (African green monkey kidney epithelial cells) and A549 cells (Human lung carcinoma epithelial cells) were grown in DMEM supplemented with 5% fetal bovine serum (FBS) medium and l-glutamine under 5% CO_2_ at 37 °C. β-Escin (purity 90–95%) was purchased from Sigma-Aldrich (Product No. E1378), dissolved in dimethyl sulfoxide (DMSO) as a stock at 50 mM, and stored at − 20 °C. ZIKV (PRAVABC59, ATCC^®^ VR-1843™), DENV-1 (Hawaii strain), DENV-2 (PL046 strain), DENV-3 (H87 strain), and DENV-4 (H241 strain) were used in this study. ZIKV and DENV were propagated in Vero cells and C6/36 mosquito cells, respectively. Virus titration was determined using a fluorescent focus assay (FFA) in Vero cells.

### Cell viability assay

The cell viability of β-escin-treated cells was assessed using the Cell Counting Kit-8 assay (CCK-8, DOJINDO Laboratories, Kumamoto, Japan). Briefly, cells were seeded into 96-well plates overnight and then different concentrations of β-escin were administrated in triplicate to cells for 30 h at 37 °C. Culture medium containing 10% CCK-8 reagent was then added for 1 h. The optical density of each sample was measured at 450 nm using an ELISA reader. The values were normalized with that of untreated cells.

### Fluorescent focus and immunofluorescence assays

The fluorescent focus assay (FFA) is a highly sensitive method to determine virus titer in which a virus exhibits growth or infectious ability. Viral titers were presented in terms of fluorescent focus units (FFU). Briefly, virus solutions were serially diluted tenfold and added to a monolayer of confluent Vero cells in 12-well plates for 2 h adsorption. DMEM containing 1.2% methylcellulose and 2% FBS were added to the cells for 2 days after which the cells were washed twice with PBS. The infected cells were fixed with 4% paraformaldehyde for 1 h and subsequently fixed with a methanol-acetone mixture for 10 min. The cells were incubated with anti-flavivirus envelope (4G2) as a primary antibody followed by staining with Alexa Fluor 488-conjugated goat anti-mouse IgG as a secondary antibody. The green fluorescent plaques were observed as virus-infected cells and counted under an inverted fluorescence microscope. The immunofluorescence assay (IFA) was done using a similar protocol as above to determine virus infection ability. Briefly, compounds and virus solutions (MOI = 0.01) were simultaneously added to cells in 12-well plates at 37 °C for 48 h. The procedure of overlaying with 1.2% methylcellulose in the former process was omitted. The cell fixation, cell penetration, and antibody staining steps were consistent with that of the FFA assay.

### Time-of-addition assay

Vero cells were infected with ZIKV (MOI = 0.2) or DENV (MOI = 1) for 2 h. β-Escin (30 μM) was added at distinct time points relative to the infection period as follows: pre-treatment (pre; 1 h prior to virus infection), co-treatment (co; during the 2 h adsorption), post-treatment 0 h (post-0 h; adding β-escin immediately after the 2 h adsorption,) post-treatment 6 h (post-6 h; adding β-escin at 6 h post-infection), post-treatment 12 h (post-12 h; adding β-escin at 12 h post-infection), full-treatment (full; during virus infection to the end). After 24 h (for ZIKV) or 40 h (for DENV), total RNA from the treated cells were extracted and the viral RNA levels were measured by qRT-PCR.

### Quantitative reverse transcription PCR (qRT-PCR)

Total RNA was extracted using Trizol reagent (Bioman, TRI200). Viral RNA levels were determined using a One-Step 2 × RT-qPCR mix SYBR Green kit (Bioman, QRP001). The primers used to detect ZIKV, DENV-1, DENV-2, DENV-3, DENV-4, and β-actin were as follows: M of ZIKV: forward primer 5′-TTGGTCATGATACTGCTGATGC-3′ and reverse primer 5′-CCTTCCACAAAGTCCCTATTGC-3′, 3′UTR of DENV-1/DENV-2/DENV3: forward primer 5′-AAAGACCAGAGATCCTGCTGTCT-3′ and reverse primer 5′-TTCTGTGCCTGGAATGATGCTG-3′, 3′UTR of DENV-4: forward primer 5′-AAAGACCAGAGATCCTGCTGTCT-3′ and reverse primer 5′-TCTGTGCCTGGATTGATGTT-3′, β-actin: forward primer 5′-AGGCACCAGGGCGTGAT-3′ and reverse primer 5′-GCCCACATAGGAATCCTTCTGAC-3′. The samples were measured in triplicate on a Roche LightCycler 480. Data were analyzed by the 2^−ΔΔCt^ method. In the DENV qRT-PCR assay, the primers used to target the 3′UTR of dengue virus serotypes 1–4 because the 3′UTR of the viral genome is highly conserved regions between flaviviruses^45^.

### Western blot analysis

Cell lysates were collected using RIPA buffer. After electrophoresis and protein transfer, immunoblotting was performed. Anti-flavivirus envelope antibodies 4G2 and anti-GAPDH antibodies were used as primary antibodies, and HRP-conjugated anti-mouse or anti-human IgG were used as secondary antibodies. The images of the bands were detected using ECL reagent and a chemiluminescence instrument.

### Binding assay

Viral solutions (MOI = 0.5) and β-escin (30 μM) were simultaneously added to cells in 12-well plates and incubated at 4 °C for 1 h. After discarding the supernatants and washing cells with PBS, cellular RNA was collected for quantification by qRT-PCR.

### Entry assay

Virus solutions (MOI = 0.5) were added to cells in 12-well plates and incubated at 4 °C for 1 h. Medium with or without β-escin (30 μM) was added after the supernatants were discarded and the treated cells were incubated at 37 °C for 1 h. Afterwards, the RNA was collected for quantification by qRT-PCR.

### Inactivation assay

The anti-ZIKV activity of β-escin was assessed under cell-free conditions. Virus stocks (2 × 10^6^ FFU) were mixed with the indicated concentrations of β-escin at 37 °C for 2 h. To avoid residual compounds affecting the result, the solutions were first diluted 100-fold. Then, the remaining infectivity of the virus titers was determined by FFA.

### Statistical analysis

The data were analyzed using GraphPad prism software and the values were expressed as the mean ± standard deviation. The statistical significance of the data was determined using a One-way ANOVA analysis with Dunnett’s multiple comparisons and p-values < 0.05 were considered statistically significant.

**Figure 6:**
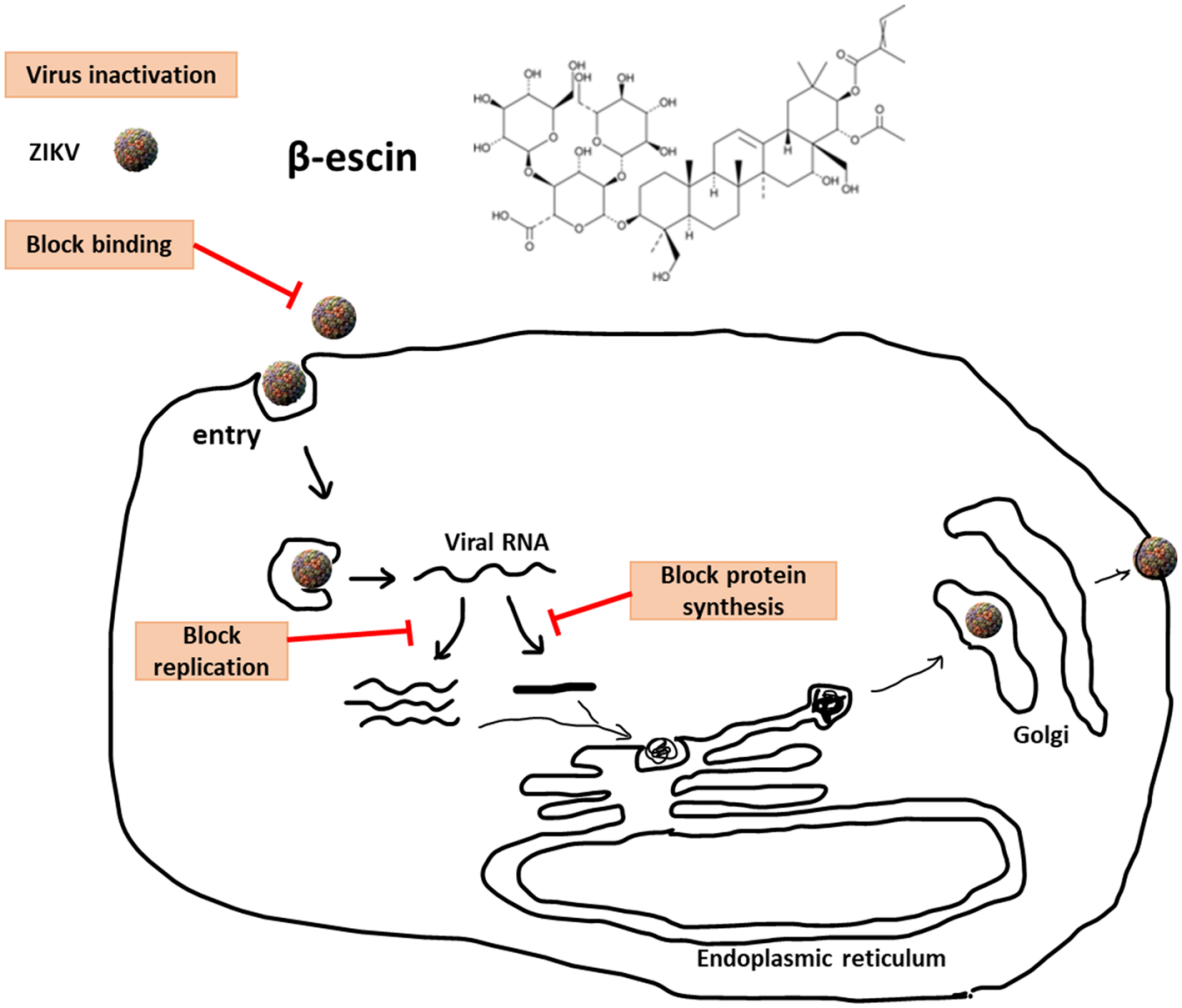
Putative antiviral mechanism of β-escin. β-escin can directly reduce virion stability in a cell-free incubation and inhibited ZIKV infection by disrupting viral binding and replication.

## Supplementary Information

Supplementary Figures.

## Data Availability

All relevant data are within the manuscript and its supplementary material.
