# Supplementary material for: Inhibitory effect of β-escin on Zika virus infection through the interruption of viral binding, replication, and stability
Source: Sci Rep. 2023 Jun 20;13:10014. doi: 10.1038/s41598-023-36871-1 (PMC10282019; doi:10.1038/s41598-023-36871-1)

Inhibitory Effect of β-Escin on Zika Virus Infection through the Interruption of Viral Binding, Replication, and Stability

Zheng-Zong Lai[1,2,3], Hsin-Hsuen Shen[2], Yen-Mei Lee[2,*]

1 Graduate Institute of Medical Science, National Defense Medical Center, Taipei 114, Taiwan
2 Department and Graduate Institute of Pharmacology, National Defense Medical Center, Taipei 114, Taiwan
3 Department of Pharmacy Practice, Tri-Service General Hospital, National Defense Medical Center, Taipei 114, Taiwan

* Corresponding author: Yen-Mei Lee
E-mail address: ymlee@mail.ndmctsgh.edu.tw (Y.-M.L.) laizengzong@gmail.com (Z.-Z.L.), hsshen@mail.ndmctsgh.edu.tw (H.-H.S.)
Tel.: +886-2-87923100 ext. 18649 (Y.-M.L.)

Figure 2E Multiple exposures

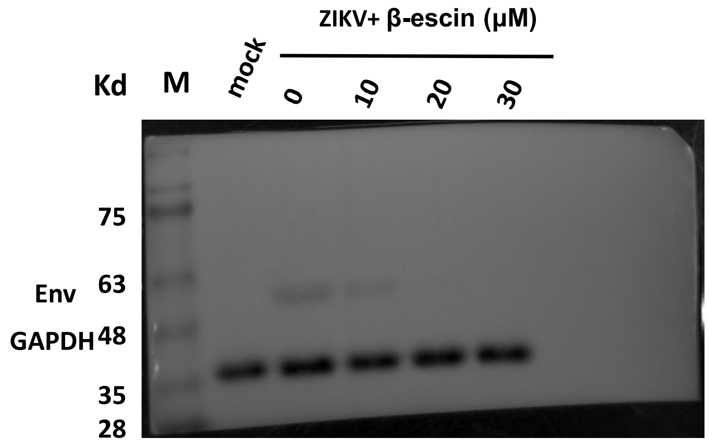

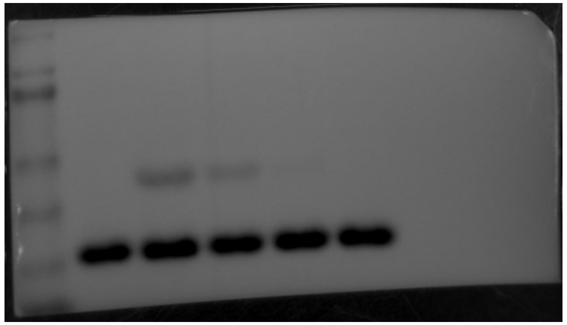

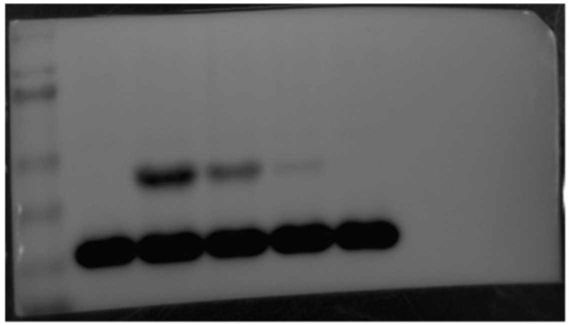

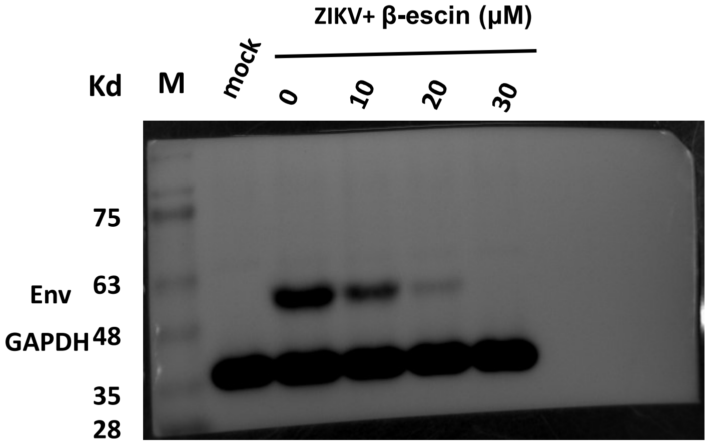

Supplement: Supplementary file 1 — Supplementary Figures. [file 41598_2023_36871_MOESM1_ESM.pdf]
